# TIAM1 promotes chemoresistance and tumor invasiveness in colorectal cancer

**DOI:** 10.1038/s41419-019-1493-5

**Published:** 2019-03-19

**Authors:** Daisuke Izumi, Shusuke Toden, Elsie Ureta, Takatsugu Ishimoto, Hideo Baba, Ajay Goel

**Affiliations:** 1 Center for Gastrointestinal Research, Center for Translational Genomics and Oncology, Baylor Scott & White Research Institute and Charles A. Sammons Cancer Center, Baylor University Medical Center, Dallas, TX USA; 2 Department of Gastroenterological Surgery, Graduate School of Medical Sciences, Kumamoto University, Kumamoto, Japan; 3 Department of Surgery, Japan Community Health Care Organization Kumamoto General Hospital, Yatsushiro, Japan; 4 The International Research Center for Medicine Sciences, Kumamoto University, Kumamoto, Japan

## Abstract

Accumulating evidence suggests that cancer cells with stem cell-like features have higher resistance to chemotherapeutic agents. Herein, we identified T-lymphoma invasion and metastasis-inducing protein-1 (*TIAM1)* as one of the Wnt-signaling associated genes which drives self-renewal and its expression is upregulated by cancer associated fibroblasts (CAFs). *TIAM1* expression was assessed in resected colorectal cancer (CRC) tissues from 300 patients who did or did not respond to chemotherapy. siRNA and CRISPR/Cas9 was used to examine whether the inhibition of *TIAM1* affects chemosensitivity of CRC. We demonstrate that stemness through Wnt signaling regulates chemosensitivity and this phenomenon occurs exclusively in cancer stem cells. Subsequently, we established patient-derived CAFs and tested whether the drug sensitivity of CRC cell lines is altered with CAF-derived conditioned medium. High-*TIAM1* expression correlated significantly with poor prognosis of CRC patients, and was overexpressed in patients who did not respond to chemotherapy. We demonstrated that the inhibition of *TIAM1* enhanced sensitivity to chemotherapeutic drugs and reduced tumor invasiveness in a series of experiments in vitro. Moreover, CAF-derived conditioned media increased stemness and chemoresistance in CRC cell lines through *TIAM1* overexpression. In addition, we validated *TIAM1* associated drug sensitivity using a xenograft model. We have demonstrated that *TIAM1* is overexpressed in CRC tumors from patients who did not respond to chemotherapeutic drugs and levels of *TIAM1* expression served as an independent prognostic factor. Mechanistically, CAFs enhanced CRC chemoresistance through *TIAM1* overexpression. Collectively, these results suggest that *TIAM1* regulates chemosensitivity in tumors and stroma and thus may be an attractive therapeutic target.

## Introduction

Colorectal cancer (CRC) is the third most commonly diagnosed cancer and the second leading cause of cancer-related deaths in the US^1^. In spite of recent advancements in CRC treatment that have led to significantly improved clinical outcomes of patients with advanced CRC, recurrence, and metastatic spread of the disease remains the major cause of mortality from this disease. While current first-line chemotherapeutic treatment for CRC, 5-fluorouracil (5-FU) in combination with oxaliplatin (L-OHP) or irinotecan (CPT-11)^2^, is generally effective, most patients eventually develop acquired resistance to these chemotherapeutic agents^3^. Therefore, it is imperative to understand the underlying mechanisms by which drug resistance develops in CRC, in order to develop effective therapeutic strategies for CRC patients who do not respond well to such treatments.

One of the key cell types associated with progression and metastasis of cancer are cancer stem cells. This subpopulation of cancer cells with stem cell-like properties, i.e., cancer cells with self-renewal, multi-differentiation, and tumor initiation capabilities, are responsible for acquisition of drug resistance^6,7^. Interestingly, cancer stem cells have been shown to have high Wnt activity^8^. Cancer cells with a high expression of stem cell markers, including Oct-4 and Nanog, have increased resistance to chemotherapeutic drugs than low or non-expressing cells^9–12^. Furthermore, the Wnt-signaling pathway has been identified as one of the key pathways that drives self-renewal in CRC^13^ and many CRCs carry at least one mutation in a gene in the Wnt-signaling pathway, rendering the pathway constitutively active. Thus it is not surprising that Wnt signaling enhances drug resistance, but the therapeutic targeting of genes in the Wnt-signaling pathway presents multiple challenges due to its complexity. Nevertheless, recently one of the Wnt-responsive genes, *TIAM1*, a guanine nucleotide exchange factor specific for Rac1, was shown to be overexpressed in human colonic adenomas and mouse intestinal tumors^14^, and found to act as oncogene through increased cellular invasiveness and promote epithelial-mesenchymal transition (EMT) in multiple cancers^15–18^. Moreover, the suppression of *TIAM1* retards cell growth, colony formation, invasion, and migration potential in CRC^19,20^.

The stromal microenvironment within cancers is another integral factor which influences the therapeutic response of cancer^21,22^. While the tumor-associated stroma is composed of various cell types and extracellular components, cancer-associated fibroblasts (CAFs) have been identified as the key stromal cells which regulate tumor progression^23^. CAFs are known to advance tumor growth, angiogenesis, metastasis, and produce resistance of tumor cells to chemotherapy. A number of different signaling pathways have been shown to be involved in this process, including the Wnt-signaling pathway^5^. Therefore, once again the Wnt-signaling pathway appears to be a major signaling pathway that can lead to activation of CAFs^24^, and subsequently promote drug resistance in tumors^25,26^. Herein, using a series of in vitro, animal studies and clinical patient cohorts, we for the first time report that *TIAM1* is a key Wnt-signaling gene that is frequently overexpressed in CRC, which leads to enhanced drug resistance and stemness in CRC, and a process that is tightly orchestrated by the CAFs within the tumor microenvironment in CRC.

## Materials And methods

### Patients and specimen collection

This study included an analysis of primary CRC specimens obtained from 300 patients who underwent a colectomy without preoperative treatment at the Kumamoto University Hospital, Japan, between April 2005 and June 2012. Primary CAFs were isolated from CRC tissues from the patients who underwent a colectomy at the Baylor University Medical Center from 2016 to 2017 and the purity of the CAF population was validated, as described previously^27^. The study was approved by the Institutional Review Board of the Baylor Scott & White Research Institute and the Kumamoto University, Japan. A written informed consent was obtained from all patients. Patients were defined as responders and non-responders for response to chemotherapy according to the response evaluation criteria in solid tumors (version 1.1).

### High-throughput gene expression data analysis for candidate identification and validation

For the candidate identification and discovery phase, we first analyzed the gene expression dataset of CRC cell lines (GSE10843), using the Oncomine database (https://www.oncomine.org). We assessed the correlation between genes in the dataset and Oct4, and focused on genes within the Wnt-signaling pathway. We identified six such genes (Selection criteria: *R*^2^ > 0.6, *P* *=* 0.001, log2 fold change > 2; Supplementary table), among which, *TIAM1* had the highest correlation with *OCT4* expression. To further evaluate the involvement of *TIAM1* and drug sensitivity, we obtained two independent publicly available gene expression datasets from the Gene Expression Omnibus (https://www.ncbi.nlm.nih.gov/geo/), which included therapeutic response measurement of CRC to FOLFOX (GSE28702) or FOLFIRI (GSE62080).

### RNA isolation and quantitative reverse-transcription PCR

RNA extraction from tissue samples was performed using the miRNeasy RNA isolation kit (Qiagen, Valencia, California, USA) and AllPrep DNA/RNA FFPE kit (Qiagen) following the manufacturer’s protocol. The synthesis of complementary DNA (cDNA) was performed from 500 ng of total RNA using the High Capacity cDNA Reverse-Transcription Kit (Invitrogen, Carlsbad, CA, USA). We performed quantitative real-time reverse-transcription analysis (qRT-PCR) using the SensiFAST™ SYBR® Lo-ROX Kit (Bioline, London, UK) and the Quantstudio 7 Real-Time PCR System (Life Technologies, Carlsbad, CA, USA). The average expression levels of the target genes were normalized against β-actin using the comparative CT method. To ensure consistent measurements throughout all assays, for each PCR amplification reaction, three independent RNA samples were loaded on to a plate as internal controls to account for potential plate-to-plate variation, and the results from each plate were normalized against the internal normalization controls. The expression levels of TIAM1 were divided into high and low expression groups according to the median expression values in order to investigate correlation between TIAM1 expression and chemotherapeutic response in the patient cohort.

### Cell lines

The CRC cell lines HCT116, SW480, SW620, DLD-1, HCT15, CaCO2, COLO320, HT29, and RKO were purchased from ATCC (Manassas, VA). All cell lines were grown in Iscove’s Modified Dulbecco’s Medium (IMDM) (Sigma-Aldrich, St. Louis, MO) supplemented with 10% fetal bovine serum (FBS) and 1% penicilin-streptomycin (Thermo Fisher Scientific, Waltham, MA, USA). The cells were maintained at 37 ℃ in a humid atmosphere containing 5% CO_2_.

### Antibodies and siRNA transfection

The polyclonal antibody against *TIAM1* was purchased from Bethyl Laboratories, Inc (clone A300-099A; Montgomery, TX, USA). The monoclonal antibody against β-actin was purchased from Sigma-Aldrich (Darmstadt, Germany). Antibodies against Nanog (D73G4, #4903), Oct4 (#75463, D7O5Z), ALDH (D9Q8E, #54135), phospho-Rac1/Cdc42 (Ser71, #2461), and Rac1/Cdc42 (#4651) were purchased from Cell Signaling Technology (Danvers, MA, USA).

*TIAM1* expression was transiently downregulated using two predesigned Silencer Select siRNAs directed against *TIAM1* (#s14138 and s14139) (Thermo Fisher Scientific), and a non-targeting siRNA was used as a negative control. CRC cells were transfected with the annealed siRNA for 24 h using Lipofectamine RNAimax (Thermo Fisher Scientific), and *TIAM1* expression was assessed by qPCR, 24 h following initial transfection. Tiam1 expression was examined by western blotting and subsequent experiments were conducted 48 h following the transfection.

### Stable knockdown of *TIAM1* expression in the HCT116 cell line

*TIAM1* expression was knocked down in HCT116 cells using *TIAM1* sgRNA CRISPR/Cas9 All-in-One Lentivector set (Human; Applied Biological Materials, Richmond, BC, Canada), which contained three *TIAM1*-specific target sequences (TGCGCCTCTCGCACAAGACG, CTCGCCGTCTCCATGAAAGT, and TGACAGGTGCTGAGCTGCCG) and scrambled sequences, according to the manufacturer’s instruction.

### Preparation of conditioned medium

Conditioned medium (CM) from CAFs was prepared by seeding fibroblasts (5 × 10^5^ cells/mL) into 100-mm plastic dishes with 10 mL of Dulbecco’s Modified Eagle Medium (DMEM) containing 10% FBS and then incubating the cells for three days. To obtain CM, the fibroblasts were washed with PBS and then incubated for an additional three days in 4 mL of DMEM + 2% FBS. Medium was thereafter collected from each dish and centrifuged at 1000 × *g* for 5 min at 4 °C to pellet cell debris. The supernatant was immediately used as CM. DMEM containing 2% FBS was used as a control.

### Western blotting

Cultured cells in 6-well plates were washed once in PBS and lysed in RIPA lysis and extraction buffer (Thermo Fisher Scientific) supplemented with a protease/phosphatase inhibitor cocktail (Thermo Fisher Scientific). Protein samples were subjected to sodium dodecyl sulfate polyacrylamide gel electrophoresis (SDS-PAGE) and transferred to nitrocellulose membranes. The membranes were blocked with 5% low-fat dry milk in TBST [25 mM Tris (pH 7.4), 125 mM NaCl, 0.4% Tween] and thereafter incubated with the primary antibodies at 4 ℃ overnight. Signals were detected after incubation with secondary antibodies at room temperature for one hour by using ChemiDoc (Bio Rad, Hercules, CA, USA). The results were quantified using image J software (National Institutes of Health, Bethesda, MD, USA) and depict relative expression values normalized to reference protein in each image.

### Cytotoxicity and invasion assays

For cytotoxicity assays, CRC cells transfected with TIAM1 siRNA or scrambled control were plated in 96-well microtiter plates (3 × 10^4^ cells per well) and cultured for 48 h with various chemotherapeutic agents. The number of viable cells was determined using an 3-(4,5-Dimethylthiazol-2-yl)-2,5-diphenyltetrazolium bromide (MTT) assay as described previously^28^. All experiments were conducted in triplicates. Optical density was determined using the Infinite 200 Pro multi-reader and i-control 1.10 (Tecan Group Ltd, Mannedorf, Switzerland).

Cell invasion assays were performed using a BD BioCoat Matrigel Invasion Chamber (BD Biosciences, San Jose, CA, USA) according to the manufacturer’s protocol. Briefly CRC cells were seeded (HCT116, 5 × 10^4^; SW480 and SW620, 1 × 10^5^) into the upper chamber of the insert in 500 μL of serum-free medium while 750 μL of 10% FBS-containing medium was placed into the lower wells. After 22 h of incubation (37 ^o^C, 5% CO_2_), the invading cells were fixed and stained. The number of invading cells in five predetermined fields (100×) were counted using a microscope. The mean of the number counted in each of the five fields was defined as the sample value. All experiments were performed in triplicate.

### Sphere formation assays

CRC cells were dissociated into single cells and 1 × 10^3^ cells were seeded in a Costar ultra-low attachment 96-well plates (Sigma-Aldrich), in serum-free stem cell medium. Cells were treated with siRNA against *TIAM1* and corresponding control 24 h before seeding. Spheres were counted using a light microscope (Olympus, Tokyo Japan) following 5-day incubation.

### In vivo tumor formation and cytotoxicity assays

Male athymic nude mice were obtained from Envigo (Houston, TX, USA) at 5 weeks of age and kept under controlled conditions (12 h light and dark cycles). The animal protocol was approved by the Institutional Animal Care and Use Committee, of the Baylor & Scott and White Research Institute. Xenograft tumors were generated using the HCT116 cell line with stably knocked down *TIAM1* or its scrambled controls. Cancer cells were suspended in PBS and Matrigel (Corning; 1:1 ratio) and 1 × 10^6^ cells were subcutaneously injected into the back of each mouse. Matrigel was used to improve the attachment and differentiation of the cells. Twenty mice were used in each group, and subcutaneous tumors were monitored for 21 days following injection. In each group, ten mice were injected with 50 mg/kg of 5-FU every three days, and ten mice in the control group were injected with DMSO at the same frequency. The tumors were measured every two days. Tumor size was measured using calipers and the volume was calculated using the following formula: *L* × *W*^2^ × 0.5, where *L* represents length and *W* width. At 21 days post-injection, all animals were sacrificed. Tumor samples were dissected and stored in RNA-*later* (MilliporeSigma, St. Louis, MO, USA).

### Statistical analysis

All statistical analyses were performed using Medcalc V.12.3.0 (Broekstraat 52, 9030; Mariakerke, Belgium) and the GraphPad Prism V5.0 (GraphPad Software, San Diego, California, USA). The Wilcoxon signed-rank test, Mann–Whitney *U* test, and Kruskal–Wallis test were used to analyze mRNA expression data obtained from qPCR experiments, and the results were expressed as means ± SE. All *p*-values less than 0.05 were considered statistically significant.

## Results

### *TIAM1* is frequently overexpressed in patients with chemoresistant CRC

Considering that Wnt-signaling pathway is well-recognized for its involvement in both drug resistance and stemness, we first evaluated which Wnt-signaling genes are frequently associated with stemness using publically available dataset (Supplementary table)^29^. We identified six such genes (TIAM1, POSTN, CCND2, FGF18, MYCN, SALL4), which significantly correlated with Oct4, and one of these genes, T-lymphoma invasion, and metastasis-inducing protein-1 (*TIAM1*) demonstrated the most significant correlation (*R*^2^ = 0.611). Since, *TIAM1* has recently been identified as an oncogene in multiple cancers^30,31^, we next interrogated whether *TIAM1* plays a role in mediating resistance to chemotherapeutic drugs in CRC.

**Fig. 1:**
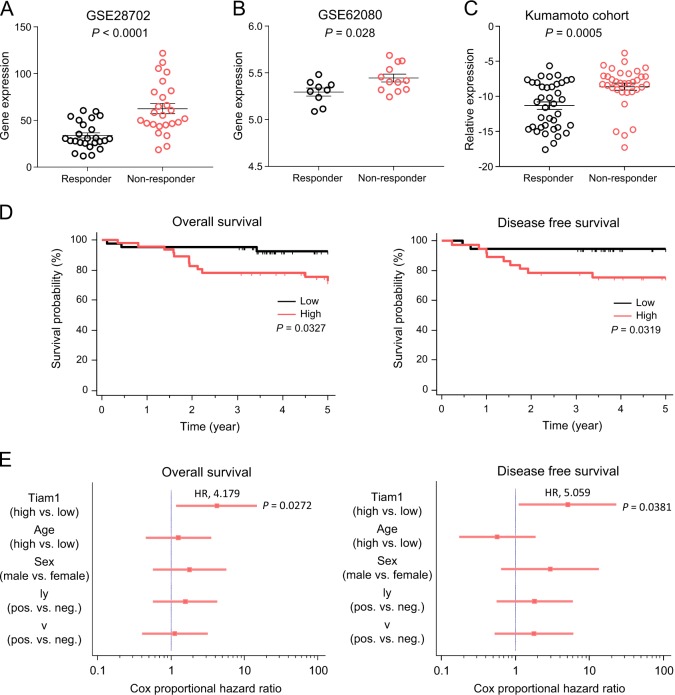
*TIAM1* overexpression in chemoresistant colorectal tumors. **a** The scatter plot illustrates the expression levels of *TIAM1* in CRC specimens in a GSE28702 dataset derived from 25 responder patients and 25 non-responder patients against FOLFOX. The *P*-value was obtained by the Mann–Whitney *U* test. **b** The scatter plot illustrates the expression levels of *TIAM1* in CRC specimens in a GSE62080 dataset derived from 9 responder patients and 12 non-responder patients against FOLFIRI. The *P*-value was obtained by the Mann–Whitney *U* test. **c** The scatter plot illustrates the expression levels of *TIAM1* in CRC specimens of the Kumamoto cohort derived from 38 responder patients and 36 non-responder patients against chemotherapy. The *P*-value was obtained by the Mann–Whitney *U* test. **d** Kaplan–Meier curves illustrate overall survival probability and disease free survival probability stratified by the *TIAM1* expression level. *P*-values were obtained by log-rank test. **e** Forest plots illustrate the Cox hazard proportional analysis based on overall survival and disease free survival stratified by the *TIAM1* expression and clinical factors

Next, to examine whether the expression of *TIAM1* correlates with therapeutic response, we investigated *TIAM1* mRNA expression in two independent CRC cohorts, which included patients that were responders or non-responders to FOLFOX (GSE28702, *n* = 50) and FOLFIRI (GSE62080, *n* = 21; Fig. 1a, b). To our surprise, the expression of *TIAM1* was significantly upregulated in patients that were non-responders to such treatments vs. those that responded in both publicly available patients cohorts (*P* < 0.05–0.001). To further evaluate the clinical significance of *TIAM1* on resistance to chemotherapeutic agents, we assessed the expression of TIAM1 in another, in-house, independent patient cohort of 72 metastatic CRC patients who underwent primary site resection followed by chemotherapy enrolled at the Kumamoto University (Kumamoto cohort). Consistent with the results from the publicly available datasets, *TIAM1* was significantly upregulated in CRC tissues from the non-responder group (*p* < 0.001; Fig. 1c). We next evaluated the clinicopathological features (age, sex, lymphatic invasion [ly] and venous invasion [v]) and prognostic significance of *TIAM1* in stage II/III patients (*n* = 110) from the Kumamoto cohort. The Kaplan–Meier analysis revealed that CRC patients with high-*TIAM1* expression demonstrated poorer survival, both overall (OS) and disease free survival (DFS), compared to those with low-*TIAM1* expression (*P = 0.0327* and *P* = 0.0319 respectively, Fig. 1d). In a Cox proportional hazard regression analysis, high-*TIAM1* expression emerged as an independent prognostic factor for poor survival (HR, 4.18; 95% CI 1.18–14.77; *P* = 0.027) and higher recurrence rate (HR, 5.06; 95% CI 1.10–23.24; *P* = 0.038; Fig. 1e) in CRC patients. Collectively, these data suggest that the high expression of TIAM1 is associated with a worse response to chemotherapeutic agents, supporting our rationale for its involvement in chemotherapeutic response in CRC.

### *TIAM1* promotes chemoresistance in CRC cells

**Fig. 2:**
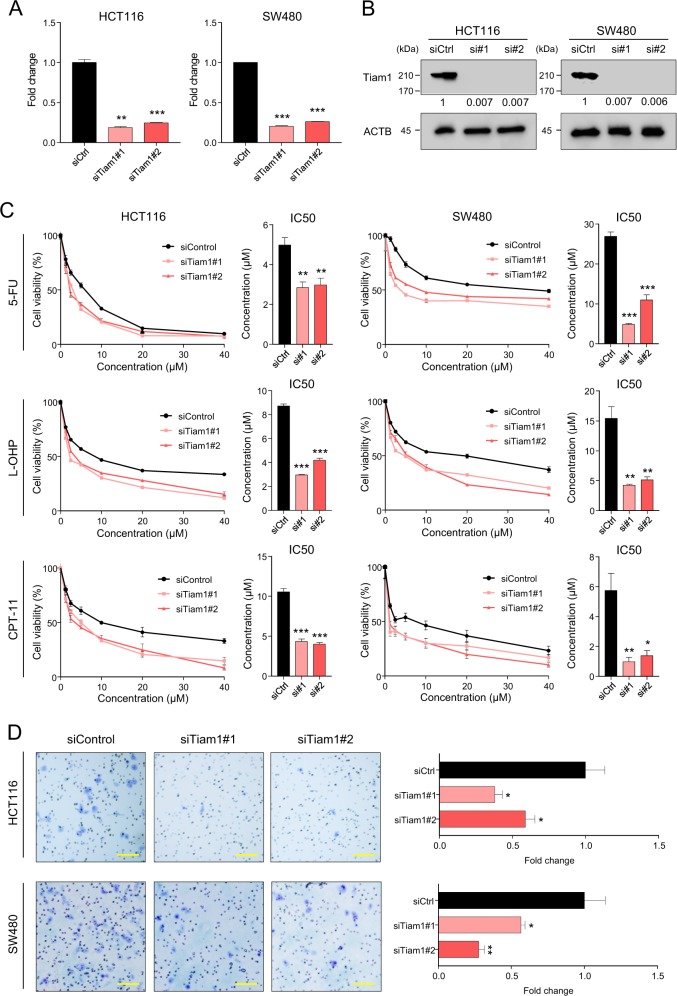
*TIAM1* promotes chemoresistance in CRC cells. **a** The bar graphs illustrate mRNA expression of *TIAM1* in CRC cell lines transfected by siRNA against *TIAM1* or the corresponding control evaluated by qRT-PCR. **b** Western blot analyses show protein expression of *TIAM1* in CRC cell lines transfected by siRNA against *TIAM1* or the corresponding control. The relative expression values normalized to reference protein are shown in each image. **c** CRC cell lines transfected by *TIAM1* siRNA or the corresponding control were incubated for 48 h with the indicated concentrations of 5-FU, L-OHP, and CPT-11 and then assayed for cell viability; the line graphs are presented as the treated/control cell ratio and the bar graphs illustrate IC50. **d** The invasion ability of CRC cell lines transfected by *TIAM1* siRNA or the corresponding control was quantified using a trans-well invasion assay. Representative microscopic fields are shown (left) and the data is presented as the treated/control cell ratio (right). Scale bars, 200 μm (×100). Error bars are presented as mean ± SE of three independent experiments, *P < 0.05; **P < 0.01; ***P < 0.001 (*T*-test)

Considering that the overexpression of *TIAM1* is associated with chemoresistant CRC in the clinical cohorts, we wanted to investigate whether *TIAM1* is involved in the regulation of drug resistance in CRC cells. We first examined the expression of *TIAM1* in several CRC cell lines to identify those with high endogenous *TIAM1* expression (Fig. S1). Since the expression of *TIAM1* was high in HCT116, SW480, and SW620 cell lines, we selected these cell lines to undertake siRNA transfection experiments. Following siRNA based in vitro experiments, suppression of *TIAM1* was confirmed at both mRNA (Fig. 2a and S1B) and protein expression levels (Fig. 2b and S1C). To investigate whether *TIAM1* regulates sensitivity towards chemotherapeutic agents used in CRC patients, we conducted cytotoxicity assays with 5-FU, L-OHP, and CPT-11 in CRC cell lines with or without *TIAM1* siRNA. In support of our hypothesis, we noted that *TIAM1* inhibition significantly enhanced sensitivity to all three chemotherapeutic agents in CRC cell lines (Fig. 2c and S1D).

One of the key characteristics of cancer cells with high resistance to chemotherapeutic agents is their higher invasion potential^32^. Therefore, next we examined invasion potential of CRC cells with or without *TIAM1* inhibition using matrigel invasion assays. As expected, the inhibition of *TIAM1* significantly decreased the invasiveness of all CRC cell lines (*p* < 0.05–0.01; Fig. 2d and S1E). To further confirm its involvement in chemoresistance, we examined *TIAM1* expression in CRC cell lines that were made chemoresistant by continuous treatment with 5-FU, L-OHP, and CPT-11 for more than a year, and established in our laboratory, as published previously^33^. In line with our other findings, intriguingly, *TIAM1* was significantly overexpressed in cell lines that were resistant against 5-FU, L-OHP, and CPT-11 when compared to parental cell lines (*p* < 0.01–0.001; Fig. S1F).

### *TIAM1* regulates chemoresistance through stemness control in CRC cells

**Fig. 3:**
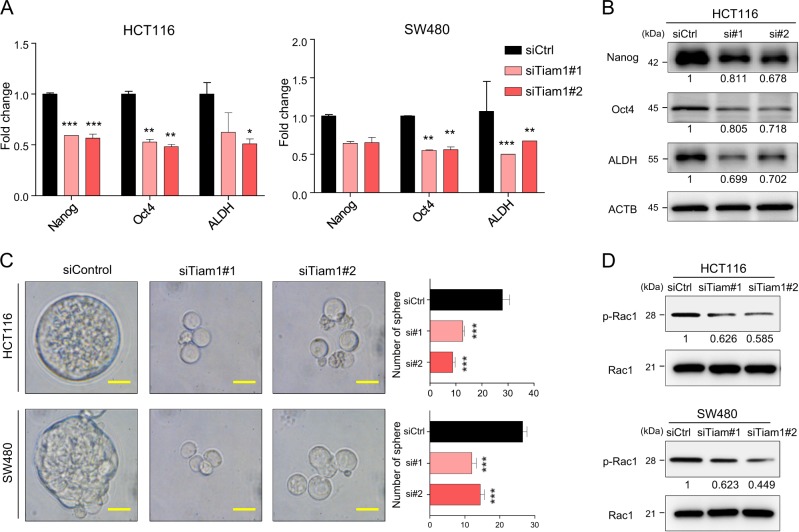
*TIAM1* regulates chemoresistance through controlling stemness in CRC cells. **a** The bar graphs illustrate mRNA expression of *NANOG*, *OCT-4*, and *ALDH* in CRC cell lines transfected with siRNA for *TIAM1* and the corresponding control. **b** The western blot analyses illustrate mRNA expression of Nanog, Oct-4, and ALDH in the HCT116 cell line transfected with siRNA for *TIAM1* and the corresponding control. The relative expression values normalized to reference protein are shown in each image. **c** A spheroid formation assay for CRC cell lines transfected by *TIAM1* siRNA or the corresponding control. Representative microscopic fields are shown (left). The image of the siCtrl is from the spheroids, and the images of the siTIAM1 are the cancer cells which did not form sphere. The bar graph is presented as the number of spheroids (right). Scale bars, 40 μm (×400). **d** The western blot analyses illustrate the protein expression of phosphorylated Rac1 and the total Rac1 of CRC cell lines transfected with siRNA for *TIAM1* and the corresponding control. The relative expression values normalized to reference protein are shown in each image. Error bars are presented as mean ± SE of three independent experiments, *P < 0.05; **P < 0.01; ***P < 0.001 (*T*-test)

Previous studies have shown that cancer stemness plays an important role in mediating chemoresistance in various cancers^6,34^. Considering that *TIAM1* suppression resulted in an improved sensitivity towards the three CRC therapeutic drugs, we hypothesized that *TIAM1* may mediate chemoresistance through regulation of cancer stemness. To investigate this further, we next examined the expression of various stemness-related genes including, Nanog, Oct-4, and ALDH in CRC cell lines treated with siRNA for *TIAM1* or the corresponding controls. As expected, these three stem cell related genes were significantly downregulated in CRC cell lines with *TIAM1* knockdown, suggesting that *TIAM1* inhibition results in decreased stemness (*p* < 0.05–0.001; Fig. 3a, b, and S2A). To investigate the interaction between *TIAM1* and stemness, we performed a sphere formation assay with cancer cells with or without *TIAM1* inhibition. *TIAM1* knockdown resulted in significant reduction of spheroid formation in numbers indicating that TIAM1 is involved in regulation of cancer stemness (*p* < 0.001; Fig. 3c and S2B). One of the key downstream targets of *TIAM1*, which has previously been shown to regulate stemness is, Rac1^35^. Rac1 activation plays a crucial role in stemness inhibited by semaphorin-3F in CRC^36^. Accordingly, we successfully confirmed that inhibition of *TIAM1* resulted in reduced phosphorylation of Rac1 (Fig. 3d and S2C). Collectively, these results indicate that inhibition of *TIAM1* suppresses cancer stemness in part through inhibition of Rac1 phosphorylation.

### *TIAM1* upregulates chemoresistance in a xenograft animal model

**Fig. 4:**
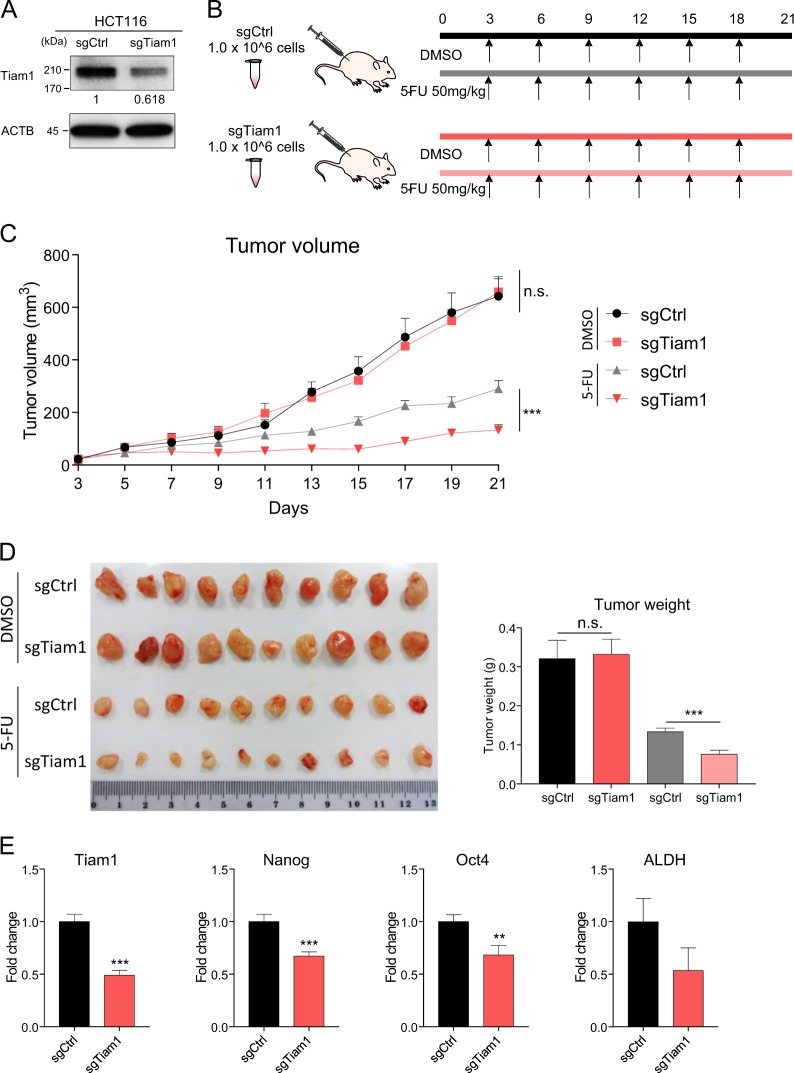
*TIAM1* upregulates chemoresistance in CRC xenograft. **a** The western blot analysis illustrates the protein expression of *TIAM1* in the HCT116 cell line stably-transfected by sgRNA against *TIAM1* or the corresponding control using CRISPR/Cas9. The relative expression values normalized to reference protein are shown in each image. **b** Overall workflow of in vivo experiments. **c**, **d** Effect of *TIAM1* knockdown in HCT116 cells on the xenograft model was assessed by evaluating tumor volume (**c**) and weight (**d**) compared to controls. **e** The bar graphs illustrate mRNA expression of *TIAM1, NANOG*, *OCT-4*, and *ALDH* in CRC tumors transfected with sgRNA for *TIAM1* and the corresponding control. Error bars are presented as mean ± SE of three independent experiments, ***P < 0.001 (Mann–Whitney *U* test)

To validate the role of *TIAM1* in mediating chemoresistance in vivo, we generated xenograft tumors in nude mice using HCT116 cells with stable knockdown of TIAM1 using CRISPR/Cas9 or the corresponding controls and subsequently treated these tumors with or without 5-FU. We first validated effective knockdown of *TIAM1* by measuring its protein expression in HCT116 cells (Fig. 4a). Furthermore, we demonstrated that these TIAM1 knockdown cells showed significant sensitization to chemotherapeutic agents in CRC cells in vitro (Fig. S3A). Twenty mice were used in each group and the tumor growth was monitored for 21 days following the initial injection of HCT116 cells. In each group, ten mice were injected with 50 mg/kg of 5-FU every 3 days, and DMSO was used as controls, as illustrated in the experimental schematic (Fig. 4b). Although no significant differences were observed between the growth of *TIAM1* knockdown and controls in DMSO treated animals, tumor size of *TIAM1* knockdown tumors was significantly inhibited by 5-FU treatment compared to the controls (*p* < 0.001; Fig. 4c and S3B). Consistently, tumor weight of harvested tumors confirmed that animals with *TIAM1* suppression demonstrated a superior response to 5-FU compared to controls (*p* < 0.001; Fig. 4d). Next, we assessed whether the expression of stemness-associated genes was downregulated in *TIAM1* knockdown tumors. Confirming our in vitro data, the expression of Nanog, Oct4, and ALDH were all downregulated in *TIAM1* suppressed tumors, suggesting that these *TIAM1* suppressed tumors contained a lower population of cancer stem cells (Fig. 4e).

### CRC-derived CAFs enhance drug resistance through *TIAM1* overexpression

**Fig. 5:**
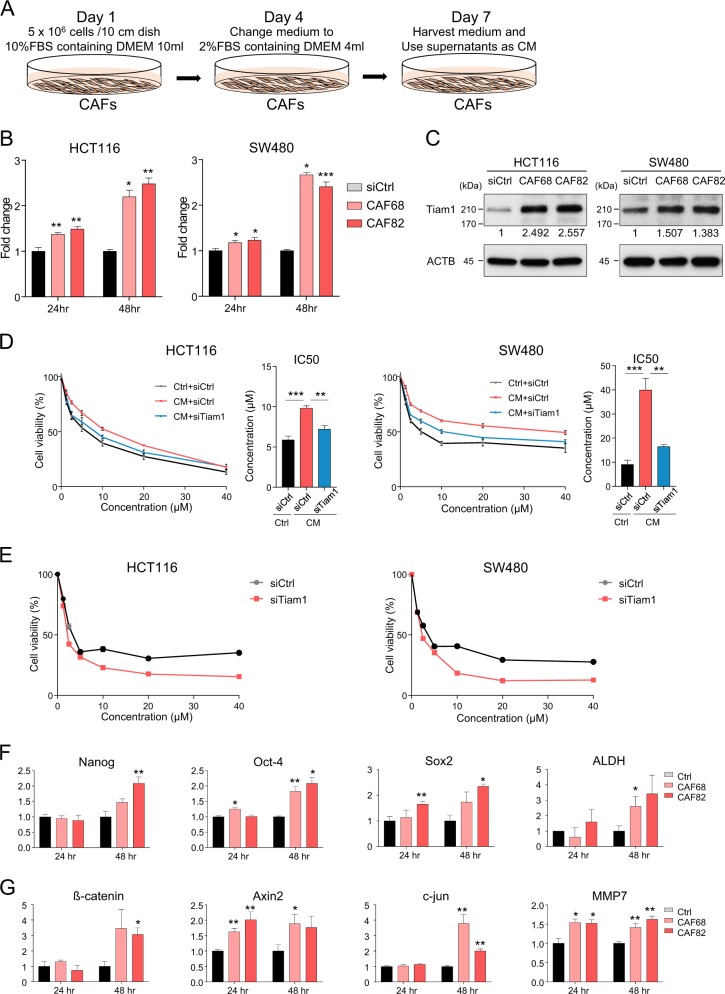
CAFs regulate chemoresistance through the upregulation of *TIAM1* expression. **a** Preparation of CM derived from CAFs. **b** The bar graphs illustrate the mRNA expression of *TIAM1* in CRC cell lines co-cultured with CAF-derived CM established from two different CRC patients for 24 or 48 h. **c** The western blotting analyses illustrate the protein expression of *TIAM1* in CRC cell lines co-cultured with CAF-derived CM established from two different CRC patients for 48 h. The relative expression values normalized to reference protein are shown in each image. **d** CRC cell lines transfected by *TIAM1* siRNA or the corresponding control were co-cultured with CAF-derived CM or the corresponding control for 48 h with the indicated concentrations of 5-FU, then assayed for cell viability; the line graphs are presented as the treated/control cell ratio and the bar graphs illustrated IC50. **e** CRC cell lines co-cultured with CM derived from CAFs, transfected by *TIAM1* siRNA or the corresponding control were incubated for 48 h with the indicated concentrations of 5-FU, and then assayed for cell viability; the line graphs are presented as the treated/control cell ratio. **f** Bar graphs illustrate mRNA expression of *NANOG*, *OCT-4, SOX2*, and *ALDH* in CRC cell lines co-cultured with CAF-derived CM or corresponding control for 48 h. **g** Bar graphs illustrate mRNA expression of Nanog, Oct-4, Sox2, and ALDH in CRC cell lines co-cultured with CAF-derived CM or the corresponding control for 48 h. Error bars are presented as mean ± SE of three independent experiments, *P < 0.05; **P < 0.01; ***P < 0.001 (*T*-test)

Emerging evidence indicates that the tumor microenvironment plays a pivotal role in acquisition of drug resistance as well as maintenance of stemness in multiple cancers, including CRC^37,38^. In particular, cancer-associated fibroblasts (CAFs) are known to enhance stemness and invasiveness in CRC^25^. Therefore, we hypothesized that CAFs contribute to overexpression of *TIAM1* in CRC, which subsequently manifests in increased chemotherapeutic resistance in cancer patients. To investigate whether CAFs contribute to *TIAM1* overexpression, we established CRC-patient-derived primary CAFs using a previously described method^27^. Subsequently, CM was harvested from these CRC-patient-derived CAFs and used for co-culture experiments (Fig. 5a). Intriguingly, *TIAM1* expression was significantly upregulated in CRC cell lines cultured with CM for 48 h (Fig. 5b, c), suggesting that CAFs stimulate TIAM1 upregulation. Next, we examined whether CAF CM can enhance the chemoresistance of CRC cell lines and whether inhibition of *TIAM1* expression in CRC cell lines by siRNA can rescue this enhancement. As expected, CAF CM increased 5-FU resistance in both CRC cell lines, but this enhancement was attenuated by *TIAM1* siRNA inhibition (Fig. 5d). Consistent results were obtained for  CPT-11 and L-OHP (Fig. S4A). Collectively, these data suggest that colorectal CAFs enhance chemoresistance through *TIAM1* overexpression. Furthermore, we examined whether direct inhibition of *TIAM1* in CAFs can inhibit CAF CM-induced chemoresistance. Intriguingly, inhibition of *TIAM1* in CAFs (Fig. S4B and C) resulted in enhanced sensitization of 5-FU to CRC cells compared those cultured with control CAFs (Fig. 5e). In addition, we investigated whether colorectal-CAF-derived CM-induced chemoresistance is associated with stemness regulation by assessment of CRC stemness markers as well as Wnt-signaling regulating genes. Surprisingly, the expression of Oct4, Nanog, Sox2, and ALDH were upregulated in CRC cell lines treated with CM of CAFs (Fig. 5f), while Wnt associated genes were also upregulated in CRC cell lines treated with CAF-derived CM (Fig. 5g). Collectively, these data suggest that CAFs promote chemoresistance through enhancement of stemness as well as Wnt-signaling activation.

## Discussion

Identification of the potential therapeutic targets for drug resistance is critical to improve the outcome of CRC patients. In the present study using a series of in vitro and in vivo experiments, we have demonstrated for the first time that *TIAM1* regulates drug sensitivity of CRC chemotherapeutic agents. Furthermore, cross-drug sensitization of *TIAM1* inhibition corresponded with suppressed stemness. To further highlight the importance of *TIAM1* expression in CRC, we showed that *TIAM1* was overexpressed in CRC tissues derived from patients who did not respond to chemotherapy compared to those responded to the treatments. Finally, we demonstrated that colorectal CAF-derived CM overexpressed *TIAM1* expression and enhanced resistance to chemotherapeutic agents in the CRC cell lines and inhibition of *TIAM1* expression in CAFs can attenuate this process. Collectively, we have demonstrated the role of *TIAM1* in CAF-induced chemoresistance in CRC.

We have demonstrated that *TIAM1* regulates stemness-associated genes and enhances stemness of CRC cells, the underlying regulatory mechanisms appear to be complex and require further investigation. One of the potential mechanisms of *TIAM1*-induced enhancement of cancer stemness is through Rac activation^43^. Consistent with data from a previous study, we have demonstrated that *TIAM1* activates Rac in CRC^44^. However, further investigation is required to fully understand the effects of *TIAM1* inhibition on other signaling pathways. Moreover, we have demonstrated that suppression of *TIAM1* expression in the CAFs resulted in suppression of drug resistance through CAF-derived CM, this suggests that *TIAM1* plays a major role in regulation of soluble factor secretion. Several studies have recently demonstrate that fibroblast-derived exosomes play an important role on oncogenesis^25,45^. It is plausible that CAFs may transfer *TIAM1* to target cells via packaging mRNA into exosomes. Nevertheless, our findings suggest that therapeutic targeting of stromal cells could effectively attenuate chemoresistance of cancer cells.

In the present study we investigated whether *TIAM1* can be used as a potential therapeutic target not only for cancer cells, but surrounding stromal cells. Accordingly, we examined the interaction between CRC cells and CAFs on drug resistance through regulation of cancer stemness^38^. The tissue microenvironment is a complex network of intercellular interactions mediated by physical attachment, biochemical signals, and soluble molecules^39,40^. Therefore, studying signals derived from tumor microenvironment on cancer progression is challenging. Emerging evidence indicates that CAFs are one of the critical components of tumor microenvironment which interferes with drug sensitivity^41,42^. We demonstrate that CAF-derived CM resulted in overexpression of *TIAM1* in CRC cells. Moreover, inhibition of *TIAM1* expression in CAFs inhibit drug resistance and highlights the importance of suppressing *TIAM1* expression in both cancer cells and CAFs. Based on our experimental data, we showed that CM collected from CAFs with reduced expression of TIAM1 resulted in enhanced chemosensitivity, suggesting existence of a mechanism by which CAFs exert oncogenic effects in cancer cells through TIAM1. At the same time, we identified that TIAM1 expression in cancer cells was significantly higher, which could in turn upregulate the expression of TIAM1 in the CAFs. Considering that this proposed feedback mechanism is intriguing, we are currently exploring this mechanism in ongoing and planned studies.

In summary, we have demonstrated that *TIAM1* regulates resistance to chemotherapeutic agents through enhancement of stemness and our clinical data supports the importance of *TIAM1* in CRC drug resistance. Furthermore, we have demonstrate that CAFs induce *TIAM1* overexpression in CRC cell line and in part enhance drug resistance. Our study indicates that *TIAM1* is a potential therapeutic target which could target both tumor cells and CAFs. Further understanding of underlying mechanisms could lead to new therapeutic strategies for CRC treatment.

## Supplementary information

Supplementary Figure legends

Supplementary Figures

Supplementary Table
