# Supplementary material for: TIAM1 promotes chemoresistance and tumor invasiveness in colorectal cancer
Source: Cell Death Dis. 2019 Mar 19;10(4):267. doi: 10.1038/s41419-019-1493-5 (PMC6425043; doi:10.1038/s41419-019-1493-5)
Supplement: Supplementary file 1 — Supplementary Figure legends [file 41419_2019_1493_MOESM1_ESM.docx]

**Supplementary Figure Legends:**

Supplemental figure 1. (A) The bar chart illustrates the mRNA expression of *TIAM1* in CRC cell lines. (B) The bar graph illustrates the mRNA expression of *TIAM1* in the SW620 cell line transfected by siRNA against *TIAM1* or the corresponding control evaluated by qRT-PCR. (C) A western blot analysis showed the protein expression of *TIAM1* in the SW620 cell line transfected by siRNA against *TIAM1* or the corresponding control. (D) The SW620 cell line transfected by *TIAM1* siRNA or the corresponding control was incubated for 48 hours with the indicated concentrations of 5-FU, L-OHP, and CPT-11, then assayed for cell viability; the bar charts illustrate IC50. (E) The invasion ability of the SW620 cell line transfected by *TIAM1* siRNA or the corresponding control was quantified using a trans-well invasion assay. Data is presented as the treated/control cell ratio. (F) The bar charts illustrate the mRNA expression of *TIAM1* in chemo-resistant CRC cell lines and their corresponding parental CRC cell lines. Scale bars. Error bars are presented as mean ± SE of three independent experiments, **P*<0.05; ***P*<0.01; ****P*<0.001 (T-test).

Supplemental figure 2. (A) The bar chart illustrates the mRNA expression of *NANOG*, *OCT-4,* and *ALDH* in the SW620 cell line transfected with siRNA for *TIAM1* and the corresponding control. (B) A spheroid formation assay for the SW620 cell line transfected by *TIAM1* siRNA or the corresponding control was quantified. Data is presented as the treated/control cell ratio. (C) A western blot analyses illustrate the protein expression of phosphorylated Rac1 and total Rac1 of the SW620 cell line transfected with siRNA for *TIAM1* and the corresponding control. Error bars are presented as mean ± SE of three independent experiments, **P*<0.05; ***P*<0.01; ****P*<0.001 (T-test).

Supplemental figure 3. (A) The HCT116 cell line stably-transfected by *TIAM1* sgRNA using CRISPR/Cas9 or the corresponding control were incubated for 48 hours with the indicated concentrations of 5-FU, L-OHP, and CPT-11, then assayed for cell viability; the line graphs are presented as the treated/control cell ratio. (B) Three representative mice in each group were shown.

Supplemental figure 4. (A) CRC cell lines transfected by *TIAM1* siRNA or the corresponding control were co-cultured with CAF-derived CM or the corresponding control for 48 hours with the indicated concentrations of L-OHP and CPT-11, then assayed for cell viability; the line graphs are presented as the treated/control cell ratio. (B) The bar charts illustrate mRNA expression of *TIAM1* in CAFs transfected by siRNA against *TIAM1* or the corresponding control evaluated by qRT-PCR. (C) Western blot analyses show protein expression of *TIAM1* in CAFs transfected by siRNA against *TIAM1* or the corresponding control.
