# Supplementary material for: TIAM1 promotes chemoresistance and tumor invasiveness in colorectal cancer
Source: Cell Death Dis. 2019 Mar 19;10(4):267. doi: 10.1038/s41419-019-1493-5 (PMC6425043; doi:10.1038/s41419-019-1493-5)
Supplement: Supplementary file 2 — Supplementary Figures [file 41419_2019_1493_MOESM2_ESM.pptx]

## Slide 1
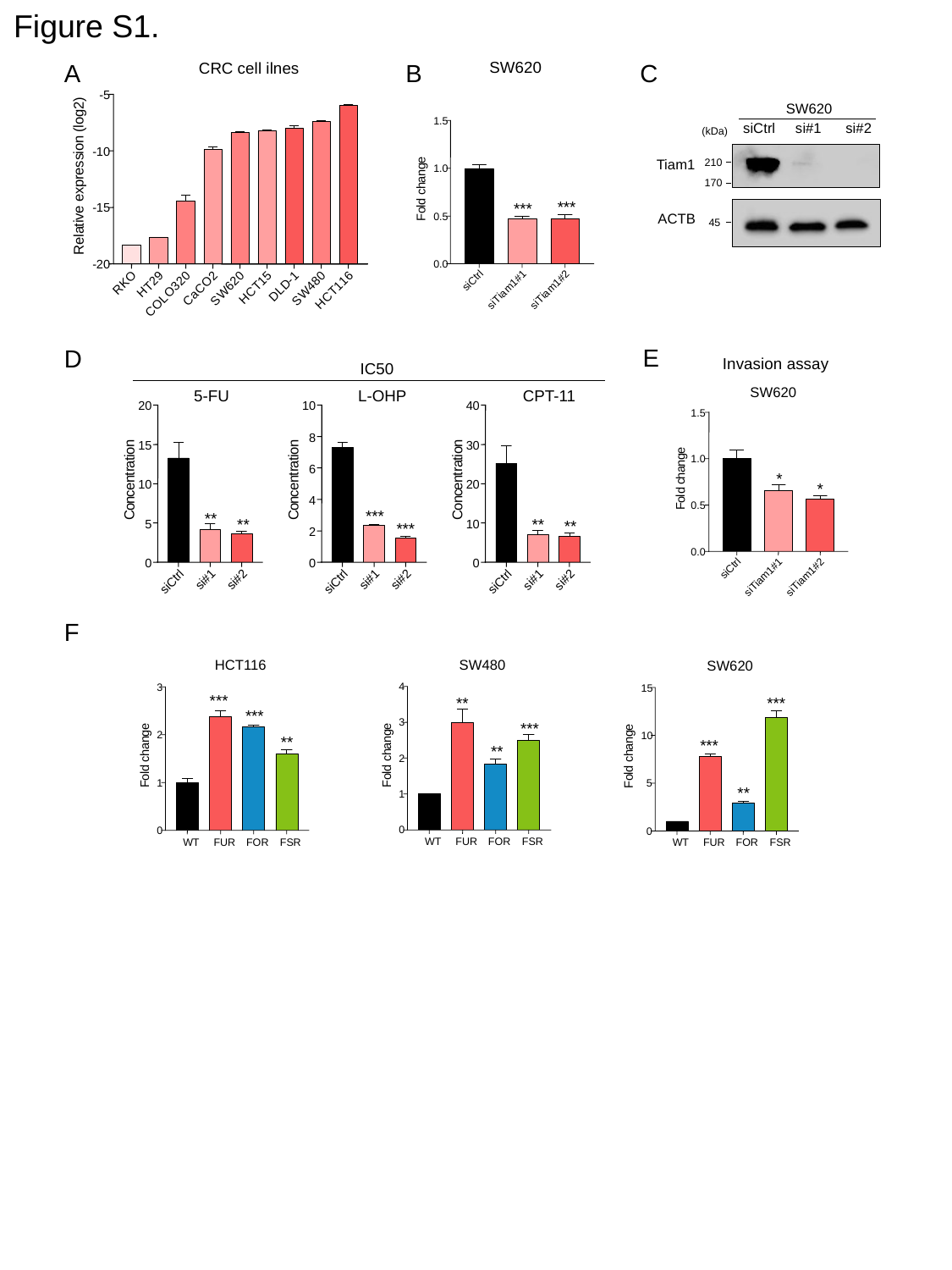

Figure S1.
A
B
SW620
C
CRC cell ilnes
-5
)
2
g
o
l
(
n
o
i
-10
s
s
e
r
p
x
e
e
-15
v
i
t
a
l
e
R
-20
5
6
9
1
0
2
0
0
O
-
2
1
1
2
2
8
O
K
D
T
1
T
3
6
4
C
R
L
T
O
C
H
W
W
a
D
C
L
H
C
S
S
H
O
C
SW620
siCtrl
si#1
si#2
1.5
(kDa)
210
Tiam1
e
g
1.0
n
170
a
h
c
***
***
d
l
o
ACTB
F
45
0.5
0.0
l
1
2
r
t
#
#
C
1
1
i
s
m
m
a
a
i
i
T
T
i
i
s
s
E
D
Invasion assay
IC50
SW620
5-FU
L-OHP
CPT-11
20
10
40
1.5
8
n
n
n
15
30
o
o
o
e
i
i
i
g
t
t
t
1.0
n
a
a
a
6
r
a
r
r
*
t
t
t
h
n
n
n
*
c
10
20
e
e
e
d
c
c
c
l
n
n
n
o
4
F
o
o
o
0.5
***
**
C
C
C
**
**
**
***
5
10
2
0.0
l
1
2
0
0
0
r
t
#
#
C
1
1
l
l
l
i
1
2
1
2
1
2
r
r
r
s
m
m
t
t
t
#
#
#
#
#
#
a
a
C
C
C
i
i
i
i
i
i
i
i
s
s
s
s
s
s
i
i
i
T
T
s
s
s
i
i
s
s
F
SW480
HCT116
SW620
4
3
15
***
**
***
***
***
3
e
e
e
g
g
g
**
2
10
***
n
n
n
**
a
a
a
h
h
h
c
c
c
2
d
d
d
l
l
l
o
o
o
F
F
1
F
5
**
1
0
0
0
WT
FUR
FOR
FSR
WT
FUR
FOR
FSR
WT
FUR
FOR
FSR

## Slide 2
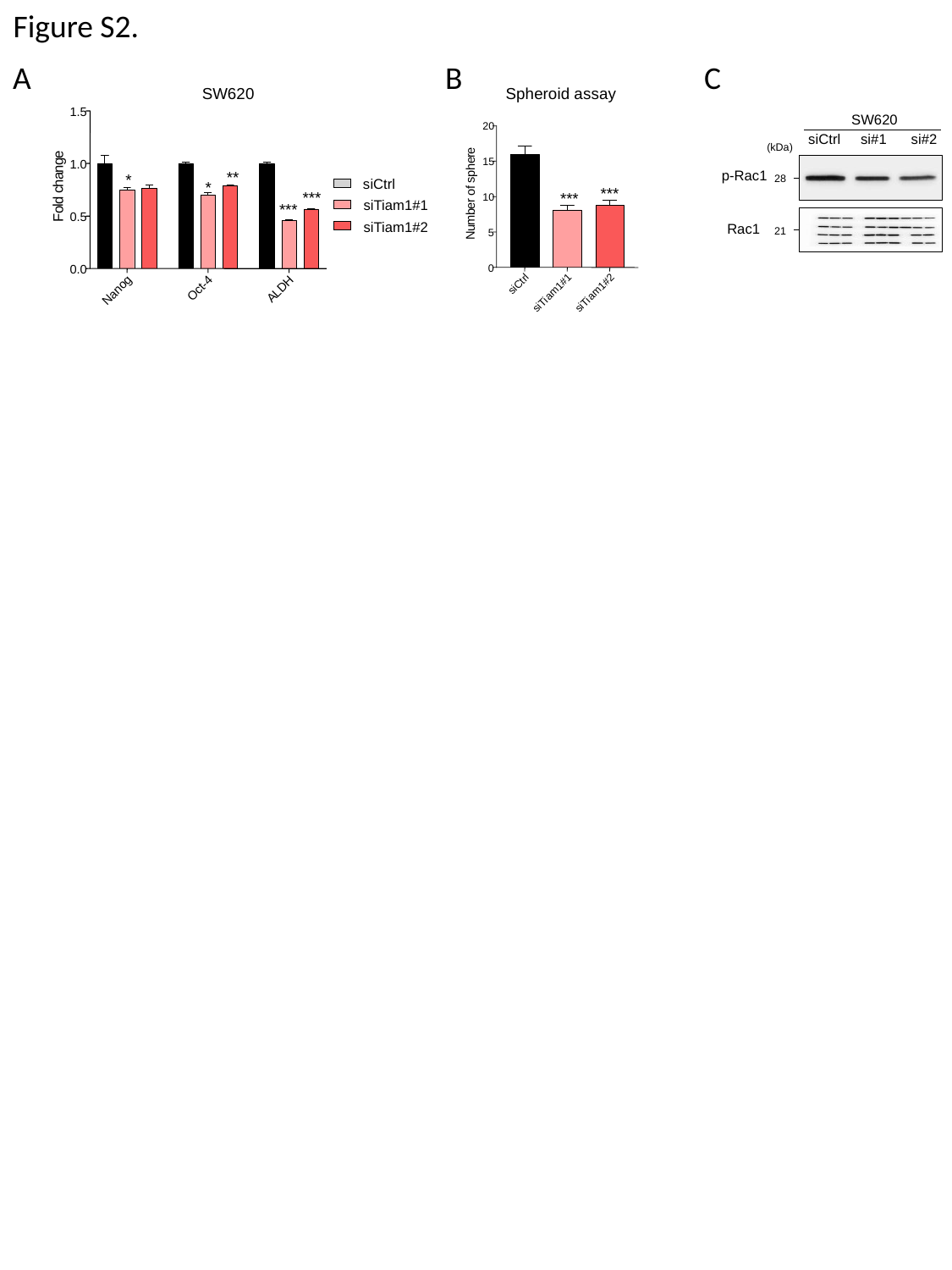

Figure S2.
A
B
C
SW620
Spheroid assay
1.5
SW620
20
siCtrl
si#1
si#2
(kDa)
e
e
r
g
e
15
1.0
h
n
p-Rac1
**
*
p
28
a
*
s
h
siCtrl
***
f
c
***
***
o
10
d
r
***
l
siTiam1#1
e
o
b
F
0.5
m
Rac1
21
siTiam1#2
u
5
N
0
0.0
l
1
2
r
g
4
H
t
#
#
-
o
C
1
1
t
D
i
n
c
s
m
m
L
a
O
a
a
A
N
i
i
T
T
i
i
s
s

## Slide 3
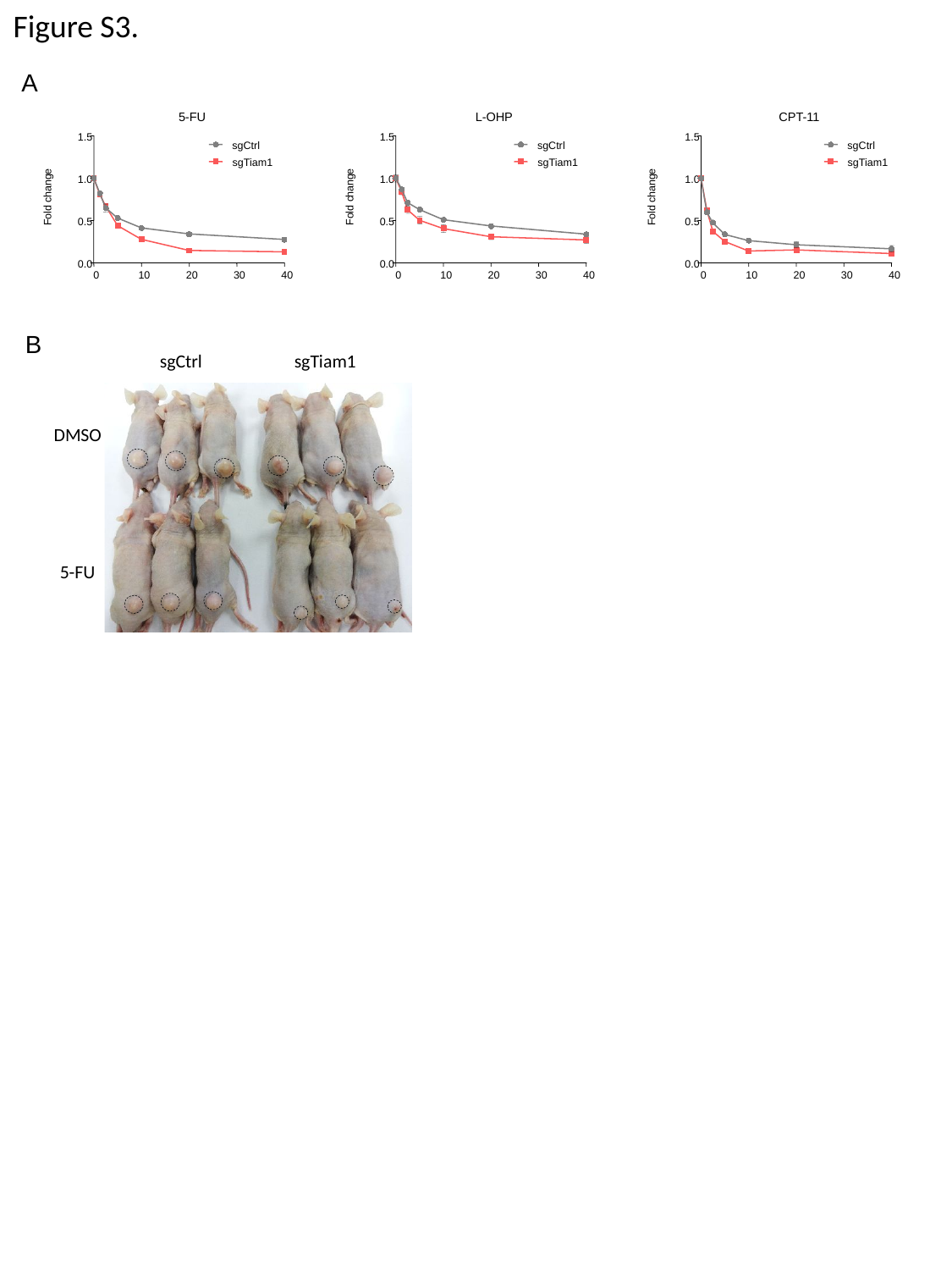

Figure S3.
A
5-FU
1.5
sgCtrl
sgTiam1
e
g
1.0
n
a
h
c
d
l
o
0.5
F
0.0
0
10
20
30
40
L-OHP
1.5
sgCtrl
sgTiam1
e
g
1.0
n
a
h
c
d
l
o
0.5
F
0.0
0
10
20
30
40
CPT-11
1.5
sgCtrl
sgTiam1
e
g
1.0
n
a
h
c
d
l
o
0.5
F
0.0
0
10
20
30
40
B
sgCtrl
sgTiam1
DMSO
5-FU

## Slide 4
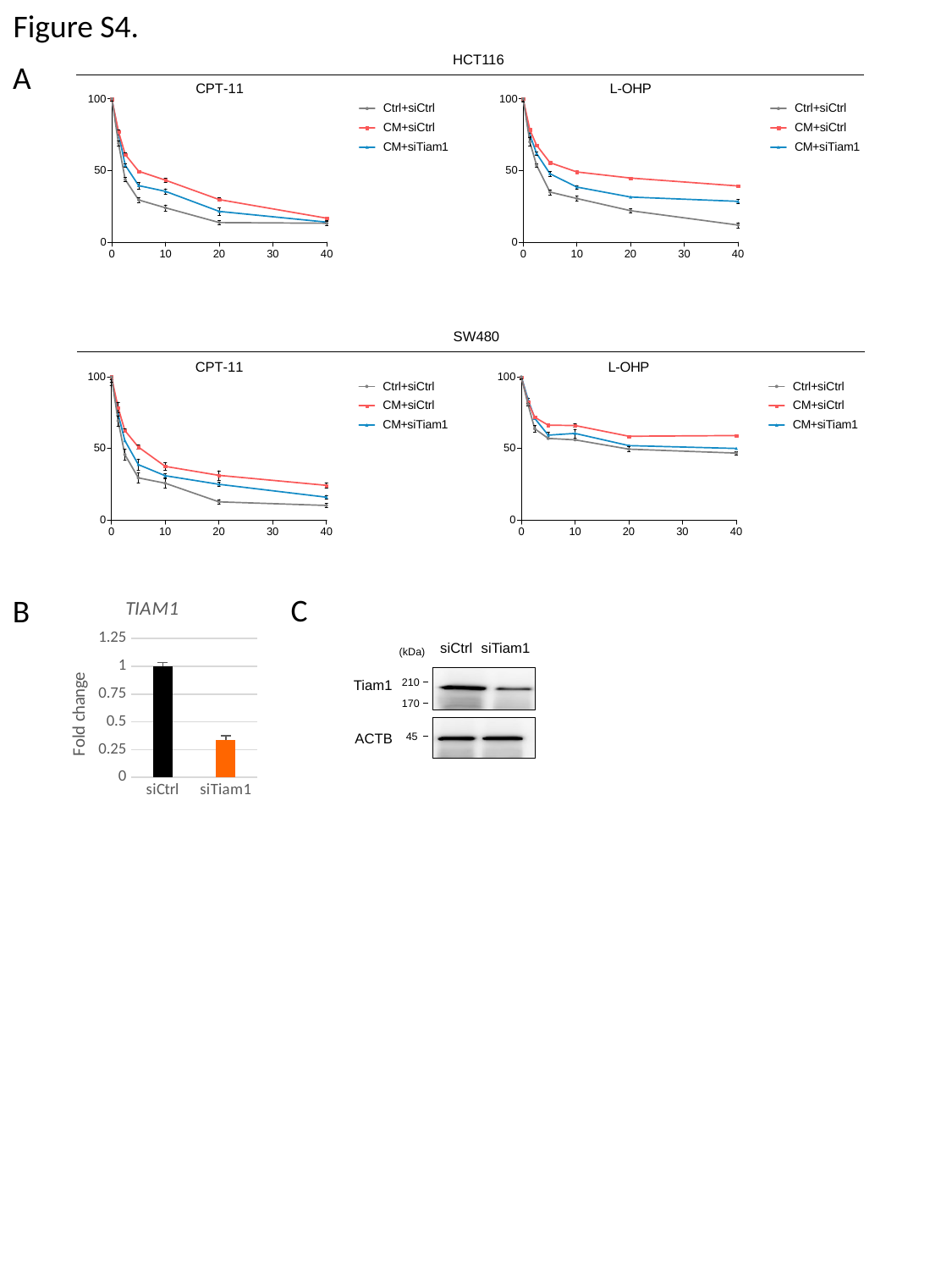

Figure S4.
HCT116
A
SW480
### Chart: TIAM1
| Category | |
|---|---|
| siCtrl | 1.0 |
| siTiam1 | 0.3337243401759531 |C
B
siCtrl
siTiam1
(kDa)
210
Tiam1
170
ACTB
45
