# Supplementary material for: TIAM1 promotes chemoresistance and tumor invasiveness in colorectal cancer
Source: Cell Death Dis. 2019 Mar 19;10(4):267. doi: 10.1038/s41419-019-1493-5 (PMC6425043; doi:10.1038/s41419-019-1493-5)
Supplement: Supplementary file 3 — Supplementary Table [file 41419_2019_1493_MOESM3_ESM.docx]

Supplemental table

| **Genes reported to be associated with Wnt signaling** | **Genes Coexpressed with Oct-4** | |
| --- | --- | --- |
| Gene name | Gene name | Correlation coefficient (R^2)^ |
| MYC | DPPA4 | 0.95 |
| MYCN | L1TD1 | 0.896 |
| CCND1 | FAM46B | 0.888 |
| CCND2 | MMP24 | 0.797 |
| CCND3 | ZNF589 | 0.783 |
| HNF1A | CRMP1 | 0.783 |
| LEF1 | OGDHL | 0.737 |
| PPARD | M6PR | 0.737 |
| JUN | LOC100128156 | 0.737 |
| fra1 | OR2C3 | 0.737 |
| Plaur | TFAP2C | 0.737 |
| MMP7 | INPP5F | 0.724 |
| Axin2 | ETV1 | 0.724 |
| NrCAM | TBCD | 0.712 |
| tcf4 | MYCL1 | 0.668 |
| gast | PAIP2B | 0.668 |
| CD44 | FGD1 | 0.668 |
| EphB | PPM1H | 0.668 |
| efnb1 | MAN1C1 | 0.668 |
| efnb2 | LRP4 | 0.668 |
| efnb3 | AKAP1 | 0.633 |
| BMP4 | VSIG10 | 0.633 |
| cldn1 | MBOAT1 | 0.633 |
| BIRC5 | SMAGP | 0.633 |
| VEGF | HTR2A | 0.633 |
| FGF18 | EPHB4 | 0.633 |
| ATOH1 | PTPN12 | 0.633 |
| Met | NFE2L3 | 0.633 |
| edn1 | AEN | 0.633 |
| MYCBP | TEAD4 | 0.633 |
| l1CAM | LDLRAP1 | 0.633 |
| Id2 | UGCG | 0.633 |
| jag1 | UPP1 | 0.633 |
| jag2 | MSTO1 | 0.611 |
| Msl1 | WDR55 | 0.611 |
| Tiam1 | SNX27 | 0.611 |
| nos2 | SLC25A44 | 0.611 |
| tert | PART1 | 0.611 |
| dkk1 | PEX5 | 0.611 |
| FGF9 | DUSP16 | 0.611 |
| LBH | ZNF263 | 0.611 |
| FGF20 | SAMHD1 | 0.611 |
| LGR5 | LMO4 | 0.611 |
| GPR49 | CREBL2 | 0.611 |
| Sox9 | TULP3 | 0.611 |
| Sox17 | ETV5 | 0.611 |
| Runx2 | DCAF4 | 0.611 |
| grem1 | ASH2L | 0.611 |
| SALL4 | TNRC6C | 0.611 |
| RANK | DNAJA2 | 0.611 |
| tnfrsf11b | ARFGEF1 | 0.611 |
| cyr61 | C17ORF63 | 0.611 |
| Sox2 | FGD4 | 0.611 |
| pttg | SLC25A16 | 0.611 |
| DLL1 | SLC16A10 | 0.611 |
| FoxN1 | MLRN1 | 0.611 |
| MMP26 | PQLC3 | 0.611 |
| nanog | MAP7D2 | 0.611 |
| snail1 | **TIAM1** | **0.611** |
| fn1 | RPUSD3 | 0.605 |
| Frizzled 7 | YRDC | 0.605 |
| fst | MDN1 | 0.605 |
| fzd7 | POLRMT | 0.605 |
| Wnt3a | STOM | 0.574 |
| isl1 | PGM2 | 0.574 |
| MMP2 | ETNK1 | 0.574 |
| sia1 | AEBP2 | 0.574 |
| BMP4 | KIAA0528 | 0.574 |
| myogenic bHLH | DUSP16 | 0.574 |
| en2 | M6PR | 0.574 |
| Xnr3 | CHST2 | 0.574 |
| gja1 | SNX27 | 0.544 |
| twin | REST | 0.544 |
| gjb30 | FBXO45 | 0.544 |
| rarg | LYPLA1 | 0.544 |
| dharma | WDHD1 | 0.544 |
| MITF | BICD1 | 0.544 |
| MMP9 | ZNF578 | 0.544 |
| Stra6 | NLRP2 | 0.544 |
| Wrch1 | LOC729082 | 0.544 |
| tnfrsf9 | HELLS | 0.544 |
| stra6 | RRP1 | 0.544 |
| enpp2 | CENPA | 0.544 |
| islr | RCC2 | 0.544 |
| efnb1 | MYBL2 | 0.544 |
| Twist1 | ZNF318 | 0.513 |
| MMP3 | LTC45 | 0.513 |
| snx9 | PNN | 0.498 |
| Tbx1 | CBX3 | 0.498 |
| Tbx3 | G2E3 | 0.498 |
| gcg2 | RBM25 | 0.498 |
| bglap | PATZ1 | 0.498 |
| Cdx1 | ZNF566 | 0.498 |
| ptgs2 | BACH1 | 0.498 |
| Irx3 | MRPLSS | 0.498 |
| Six3 | MTFR1 | 0.498 |
| neurog1 | C14ORF147 | 0.498 |
| SP5 | FBXO28 | 0.498 |
| NeuroD1 | PPHLN1 | 0.498 |
| Nkx2.2 | CBLL1 | 0.498 |
| Gbx2 | NSMAF | 0.498 |
| Cacna1g | C8ORF76 | 0.498 |
| IL6 | SOCS4 | 0.498 |
| wisp1 | NCOA1 | 0.498 |
| wisp2 | PEMT | 0.498 |
| igf2 | CCT6A | 0.498 |
| MDR1 | SARS2 | 0.498 |
| COX2 | KPTN | 0.498 |
| postn | RPL28 | 0.498 |
| TrCPb | ITPK1 | 0.498 |
| Cdc25 | ZNF710 | 0.498 |
| sFRP-2 | DERA | 0.498 |
| Pitx2 | CSNK1E | 0.498 |
| egfr | VAV2 | 0.498 |
| Eda | MFSD1 | 0.462 |
| CDH1 | DNAJB6 | 0.462 |
| krt | SCUBE2 | 0.462 |
| ovol1 | DPYS | 0.462 |
| fag1 | INPPSF | 0.462 |
| cdkn2a | CPVL | 0.462 |
| CTLA4 | COL4A3BP | 0.462 |
| mBTEB2 | AGL | 0.462 |
| FGF4 | GNL3L | 0.462 |
| il8 | TMEM170A | 0.462 |
| ret | CNNM3 | 0.462 |
| connexin43 | INO80D | 0.462 |
| vcan | PHKA1 | 0.438 |
| Tnfrsf19 | KHDC1 | 0.438 |
| Ubx | PCBP2 | 0.438 |
| wingless | PFKL | 0.436 |
| Dpp | DLG3 | 0.436 |
| en1 | PIM1 | 0.436 |
| en2 | FGFR2 | 0.436 |
| Dfrizzled2 | SIPA1L2 | 0.412 |
| ovo | PREX1 | 0.412 |
| sr | ABL2 | 0.412 |
| nmo | SNX24 | 0.412 |
|  | KIF26A | 0.412 |
|  | TCF15 | 0.412 |
|  | C7ORF10 | 0.412 |
|  | EML4 | 0.412 |
|  | ZNF71 | 0.412 |
|  | KIF7 | 0.412 |
|  | ZFP82 | 0.412 |
|  | PCOLCE2 | 0.412 |
|  | FZD8 | 0.412 |
|  | FNDC4 | 0.412 |
|  | FGGY | 0.412 |
|  | TUSC3 | 0.412 |
|  | DAPK1 | 0.412 |
|  | COL4A2 | 0.412 |
|  | SDC3 | 0.412 |
|  | CHST12 | 0.412 |
|  | **POSTN** | **0.412** |
|  | VPS37C | 0.412 |
|  | RAB11FIP1 | 0.412 |
|  | PELO | 0.412 |
|  | CSRP2BP | 0.412 |
|  | RIPK4 | 0.412 |
|  | TTC39B | 0.412 |
|  | ZNF320 | 0.412 |
|  | MIIP | 0.412 |
|  | PLEKHG3 | 0.412 |
|  | HSPC159 | 0.412 |
|  | TUFT1 | 0.412 |
|  | DTX4 | 0.412 |
|  | PRR5 | 0.412 |
|  | CORIN | 0.412 |
|  | AXL | 0.412 |
|  | SLC25A29 | 0.412 |
|  | TMEM150A | 0.412 |
|  | SGPL1 | 0.412 |
|  | ALDH16A1 | 0.412 |
|  | TLE3 | 0.412 |
|  | KCTD15 | 0.412 |
|  | INPP4A | 0.412 |
|  | STK35 | 0.412 |
|  | CMTM4 | 0.412 |
|  | ZNRF3 | 0.412 |
|  | DLG5 | 0.412 |
|  | IGSF3 | 0.412 |
|  | PTK7 | 0.412 |
|  | PTPRU | 0.412 |
|  | ZDHHC18 | 0.412 |
|  | ATP13A2 | 0.412 |
|  | SAMD1 | 0.412 |
|  | RUSC1 | 0.412 |
|  | SEMA4C | 0.412 |
|  | LRP6 | 0.412 |
|  | **CCND2** | **0.412** |
|  | **FGF18** | **0.412** |
|  | SLC5A6 | 0.412 |
|  | CPXM1 | 0.412 |
|  | NRBP1 | 0.412 |
|  | **MYCN** | **0.412** |
|  | ITM2C | 0.412 |
|  | LIN28B | 0.412 |
|  | TOMM7 | 0.412 |
|  | ZNF300 | 0.412 |
|  | EIF4EBP2 | 0.412 |
|  | BCL9 | 0.412 |
|  | PRKAB2 | 0.412 |
|  | ATN1 | 0.412 |
|  | LRP6 | 0.412 |
|  | TSPAN9 | 0.412 |
|  | TGIF2 | 0.412 |
|  | GTF2I | 0.412 |
|  | ZBTB39 | 0.412 |
|  | HUNK | 0.412 |
|  | C5ORF24 | 0.412 |
|  | LOC253842 | 0.412 |
|  | RNF2 | 0.412 |
|  | HIC2 | 0.412 |
|  | ACVR2B | 0.412 |
|  | LETM1 | 0.412 |
|  | **SALL4** | **0.412** |
|  | ITFG3 | 0.412 |
|  | HSPA4 | 0.412 |
|  | VSIG10L | 0.412 |
|  | SOX13 | 0.412 |
|  | SCAMP5 | 0.412 |
|  | APOC1 | 0.412 |
|  | APOE | 0.412 |
